# Cardiovascular effect of preeclampsia upon offspring development: Are (Pro) renin-renin receptor ((P)RR) and gender related?

**DOI:** 10.22038/IJBMS.2024.72486.15790

**Published:** 2024

**Authors:** Lourdes Graciela Baeza-Pérez, Sandra Edith Cabrera-Becerra, Rodrigo Romero-Nava, Erika Ramos-Tovar, Maria Elena Hernández-Campos, Pedro López-Sánchez

**Affiliations:** 1 Laboratorio de Farmacología Molecular, Sección de Estudios de Posgrado e Investigación, Escuela Superior de Medicina del Instituto Politécnico Nacional, Plan de San Luis y Díaz Mirón s/n, Casco de Sto. Tomás, Ciudad de México, México; 2 Laboratorio de Biología Molecular, Sección de Estudios de Posgrado e Investigación, Escuela Superior de Medicina del Instituto Politécnico Nacional, Plan de San Luis y Díaz Mirón s/n, Casco de Sto. Tomás, Ciudad de México, México; # These authors contributed eqully to this work

**Keywords:** Gender, Handle region peptide, Hypertension, Preeclampsia, (Pro)renin/renin receptor, Renin angiotensin - aldosterone system

## Abstract

**Objective(s):**

Preeclampsia (PE) is a complication of pregnancy that might increase progeny risk of cardiovascular and metabolic problems, mainly in males. Renin angiotensin aldosterone system is known to be involved. (Pro) renin/renin receptor ((P)RR) has been shown to participate in cardiovascular pathology. The aim of this work was to evaluate (P)RR expression and function upon cardiovascular and renal tissues from PE dams’ offspring.

**Materials and Methods:**

We used offspring from normal pregnant and preeclamptic rats, evaluating body, heart, aorta and kidney weight, length, and blood pressure along 3 months after birth. Subsets of animals received handle region peptide (HRP) (0.2 mg/Kg, sc). Another group received vehicle. Animals were sacrificed at first, second, and third months of age, tissues were extracted and processed for immunoblot to detect (P)RR, PLZF, β-catenin, DVL-1, and PKCα. (P)RR and PLZF were also measured by RT-PCR.

**Results:**

We found that offspring developed hypertension. Male descendants remained hypertensive throughout the whole experiment. Female animals tended to recover at second month and returned to normal blood pressure at third month. HRP treatment diminished hypertension in both male and female animals. Morphological evaluations showed changes in heart, aorta, and kidney weight, and HRP reverted this effect. Finally, we found that (P)RR, PLZF, and canonical WNT transduction pathway molecules were stimulated by PE, and HRP treatment abolished this increase.

**Conclusion:**

These findings suggest that PE can induce hypertension in offspring, and (P)RR seems to play an important role through the canonical WNT pathway and that gender seems to influence this response.

## Introduction

Pregnancy-induced hypertension comprises several alterations with common data. Preeclampsia (PE) is one of these illnesses, and it is important due to its high incidence and induction of several problems in the mother and the offspring (1). It has been reported that PE may induce severe cardiovascular problems in mother, even after delivery. In offspring, it has been associated to the development of diverse alterations including diabetes, metabolic syndrome, behavior problems, and hypertension, all of them reaching their maximum at adult ages. The study of pregnancy alterations and its effect upon appearance of illness in offspring at young ages or even adult stage, is the basis of the “disease origin in adult hypothesis”, first proposed by David Barker (2). There are different mechanisms that may participate in fetal programming: Epigenetic alterations, repression or activation of miRNAs, non-coding RNAs, and full systems alterations (3).

Cardiovascular alterations are frequent in children from PE mothers, being hypertension very common. The mechanisms that mediate fetal programming of adult hypertension are numerous (4, 5). For example, several authors have shown changes in kidney function (reduced number of nephrons), the neuroendocrine system (hypothalamic-pituitary-adrenal axis dysregulation), and the vascular system (vascular dysfunction and reduced density of arterioles and capillaries). In regards to this, Ligi *et al*., using colony-forming cells (ECFCs) isolated from umbilical cord blood from newborns, found abnormalities in the vascular and endothelial angiogenic properties of ECFCs from low birth weight (LBW) infants, compared with infants born at term and by normal delivery (6). Among systems participating in blood pressure regulation, renin angiotensin aldosterone system (RAAS) has been shown to suffer epigenetic and full system alterations during pregnancy (7, 8). The contribution of the RAAS to programmed development of hypertension in adult life implies changes in the expression of components of this system. These changes seem to depend upon age and sex(9).

It has been described that the (P)RR is a 350 amino acid protein with a single transmembrane domain with high affinity for renin and prorenin. Its binding to renin increases the catalytic efficiency of conversion of angiotensinogen into Ang I, and binding to (pro) renin would cause activation of this peptide by a non-proteolytic pathway. The ability to activate (pro) renin is explained by a flexible region in the binding domain of this peptide called “handle region”. This region is a protruding pentameric segment with well conserved sequences in several species as mouse, rat, and human. Handle region peptide (HRP) is a segment of prorrenin described by Ichihara in 2004(10). This peptide binds to (P)RR and inhibits prorrenin/renin interaction with receptor, acting as a competitive blocker of (P)RR(11). 

**Figure 1:**
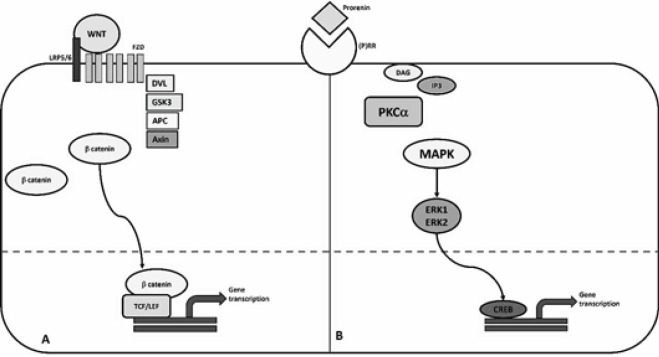
The frizzled (FZD) and LRP5/6 (lipoprotein-related receptor protein) complex binds to Wingless int-1 genes (WNT) receptor, leading to the recruitment of the disheveled (DVL) protein to the cell membrane

It is described that prorenin or renin binding to (P)RR induces an intracellular signal with phosphorylation of serine and tyrosine residues in addition to a rapid activation of the MAP kinases ERK1 (p44) and ERK2 (p42), demonstrating effects of renin and prorenin independent of those of Ang II(12). An accepted marker of this action is activation of promyelocytic leukaemia zinc finger protein (PLZF), expressed as a down-regulator molecule for (P)RR. (P)RR has also been described as a constituent part of the vacuolar ATPase V6, responsible for acidifying the intracellular medium(13) and, independently, is able to activate the WNT pathway. WNT signaling plays a key role in several physiological processes such as embryonic development, cell fate specification, and organogenesis. The pathway has been described, in fact, as two intracellular pathways: canonical and non-canonical. Canonical pathway involves participation of β-catenin complex targeted to activate several genes important in cadherin-mediated cell adhesion and synthesis of extracellular matrix components. Non-canonical pathway does not include β-catenin participation. Instead, DVL-1 may activate Ca^++^-dependent or JNK pathways, conducting to activation of effector proteins, like PKC α. Interestingly, recent studies suggest that WNT signaling is involved in the pathophysiology of atherosclerosis and, in consequence, with high blood pressure (14) (Figure 1).

We have previously shown that (P)RR seems to participate in hypertension in mothers suffering PE (7); however, it is not known if (P)RR participates in the genesis of hypertension in offspring from PE mothers and if gender influences this effect. So, the aim of this work was to evaluate (P)RR expression and possible function upon cardiovascular and renal tissues in male and female offspring from PE dams by blocking it with a selective peptide (HRP).

## Materials and Methods

Animals. All procedures described here were approved by the Animal Care Committee in our institution, according to the Mexican Official Norm (NOM-062-ZOO-1999), SAGARPA; and National Institutes of Health Guide for the Care and Use of Laboratory Animals (NIH Publications No. 8023, revised 1978, USA)

The experiments were divided in two periods: A first period using adult female rats to induce PE using the sub-renal aortic coarctation model and, a second period where only pups were used. In the first period, we used female Wistar rats weighing 250 ± 20 g with free access to tap water and a standard pellet diet (Lab Diet 5001, Purina) and kept at constant temperature (21 ± 1 °C) and humidity (50-55 %) under a 12:12 hr light/dark cycle. Animals were divided randomly in non-pregnant and pregnant groups. Subrenal aortic coarctation (SRAC), a model that resembles several human disease features, based upon indirect placental ischemia, was performed as previously described (7, 15-19). Briefly, under general anesthesia using pentobarbital (50 mg/Kg), abdominal aorta was exposed below the level of the renal arteries. After careful dissection, a silk suture, and an angled syringe gauge (22 gauge) were placed around it and progressively constricted until the syringe was tightly tied to the aorta(20). A control, sham group was also set but with no SRAC. Pregnancy was accomplished two weeks later in both groups by coupling sets of 3 female rats with a competent male per set for 48 hr; first day of pregnancy was determined when spermatozoa were observed in a vaginal smear. Both groups, normal (N=6) and SRAC animals (N=8) were allowed to deliver, progenies were wined at day 21 and pups were separated by sex. For the second period of experiments, three groups of 6 male and 6 females pups were randomly set and used at 4, 8, and 12 weeks after birth. In every period of time, body weight and size were registered, and blood pressure was measured as described below. Kidney, aorta, and heart samples were taken from every group at 4, 8, and 12 weeks of age and kept frozen to -70 °C until use. 

Pregnancy-induced hypertension model indicators in pregnant rats. Blood pressure was measured by a tail cuff method using a pressure transducer coupled to a LETICA system (Panlab, Barcelona, Spain). Animals were placed in metabolic cages (Tecniplast, Italy) to measure proteinuria in 24 hr urine samples using a commercial Bradford kit (Quick Start Bradford protein assay, Bio Rad, Hercules, Ca, USA). This parameter was expressed as g/L. Pups weights and lengths were obtained and recorded after delivery.

HRP treatments. HRP, 0.2 mg/kg/day, was administered by subdermal way during 7 consecutive days in 0.9 % saline as vehicle. The control group received same volume of 0.9 % saline. Treatments were administered during the last week of every period of time, i.e., during the 4^th^ week in the 4 weeks group, the 8^th^ week in the 8 weeks group and the 12^th^ week in the corresponding group.

Blood pressure was measured in offspring from both groups as previously described. At the end of every period of time, (P)RR, PLZF, DVL-1, β-catenin, and PKCα were measured by immunoblot and RT-PCR in tissues mentioned above.

Immunoblot assays. Protein expression was assayed by immunoblot. Experiments were performed on tissues from PE and non-PE offspring, male and female. At the end of 4, 8 and 12 weeks, animals (and corresponding time-control animals) were sacrificed with pentobarbital (65 mg/Kg), to obtain heart, aorta, and kidney. All tissues were dissected and homogenized with Tris 100 mM, pH 7.4 using a cocktail of protease inhibitors (Complete Mini, EDTA-free, Roche Diagnostics, Germany), centrifuged, and protein concentration was measured in the supernatant using Bradford´s method. 100 µg of protein of each sample were loaded in 10% SDS-PAGE under reducing conditions. Afterward, proteins in the gel were transferred to polyvinylidene fluoride (PDVF) membranes using a semi-dry electroblotting system (BIORAD, Hercules, CA, USA). Membranes were incubated with rabbit polyclonal specific antibodies against (P)RR (ATP6AP2, BIOSS, USA), PLZF, DVL-1, β- Catenin, PKC α, and β-actin (Santa Cruz Biotechnology, CA, USA). Secondary antibodies conjugated with horseradish peroxidase were used and bands were identified by chemoluminescence (Amersham International, Arlington Heights, IL, USA). Membranes were photographed and the image digitalized to carry out densitometric analysis using Quantity One software (Biorad, Hercules, CA, USA). The relative presence of each protein was normalized with β-actin as housekeeping protein.

RNA Isolation and real time PCR. Heart, aorta, and kidney from rats sacrificed at 4, 8, and 12 weeks were used (N=4). Total RNA was isolated using a standard extraction procedure (Trizol, Invitrogen, USA) and cDNA was obtained with the SuperScript II Reverse Transcriptase kit, (Invitrogen, USA). Total RNA was quantified using a spectrophotometer (IMPLEN Nanophotometer, UK), and 2 µl of RNA were used. Specific primers were designed for rat (P)RR (Fwd: 5’-GGCTCATCTCCGCTTTAGCA-3 ‘; Rev: 5’-GCACTCGACACCAGAGAAGA-3’), for rat PLZF (Fwd: 5’-AGAGCACACTCAAGAGCCAC-3’; 5’- GCTGAACTTCTTGCCACAGC-3’), and rat GAPDH (Fwd: 5’-CTACTGGCGTCTTCACCACC-3’; 5’- GGCGGAGATGATGACCCTTT-3’). All samples were analyzed by using a Techne Genius FGEN02TP Light Cycler (Techne, Cambridge, UK). Cycling conditions were: 10 sec at 95 ºC, 30 sec at 60 ºC, 10 sec at 72 ºC, and cooling of 30 sec at 0 ºC. The total amplification run was 30 cycles. The relative expression was calculated using the 2-ΔΔCT method. Expression levels of the targeted mRNAs were normalized using 18S RNA.

***Statistical analysis***

All results are expressed as mean ± SD. ANOVA followed by Bonferroni´s or Tukey test were used to compare groups using Graph Pad Prism 10 software. Differences were considered significant when *P*<0.05.

## Results

We have previously shown that SRAC induces a preeclampsia-like illness in rats. In Table 1, blood pressure and proteinuria induced in dams are shown. To study the effect of PE upon intrauterine development, we measured in all pups birth weight and size. In Table 1 these parameters are included. Mean size in normal animals was 5.8 ± 0.1 cm, and PE-derived animals mean was 4 ± 0.04 cm. On the other hand, mean body weight was 6.8 ± 0.13 g in normal pregnancy offspring, while in PE-derived offspring, mean body weight was 4.6 ± 0.17 g. Number of pups was also diminished in animals from PE, being 10 ± 0.7 pups, while in normal pups, it was 14.75 ± 0.47. Clearly, animals from preeclamptic pregnancy presented a low number of pups, weight and a small size compared to animals from normal pregnancy, which indicates that model functioned as expected.

**Figure 2:**
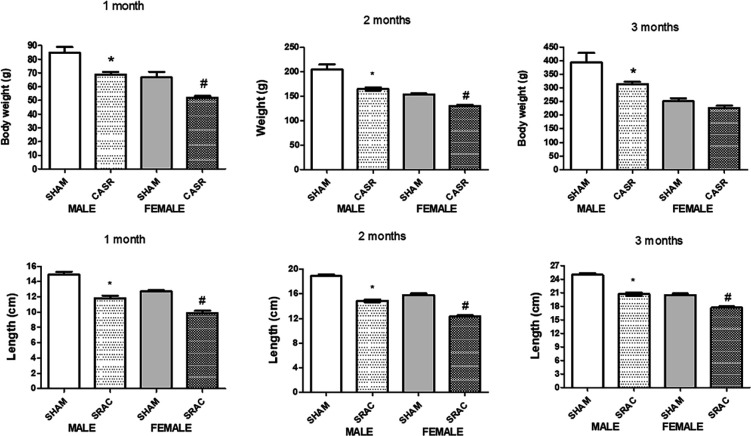
Body weight and length in offspring from normal (SHAM) and PE (SRAC) pregnancies

To also explore the role of gender in offspring development, we followed body weight for 4, 8, and 12 weeks after birth, both in male and female animals. In Figure 2 we show body weight at these times. In 1 month-old offspring, there were normal differences between males and females from normal pregnancy animals. Body weight in PE-derived animals, in contrast, was lower than control animals.

Interestingly, at 12 weeks after birth, only males from PE-pregnancies remained with lower body weight compared to respective control animals. Female animals from PE recovered body weight, and there were no differences compared to their corresponding control group. 

**Figure 3:**
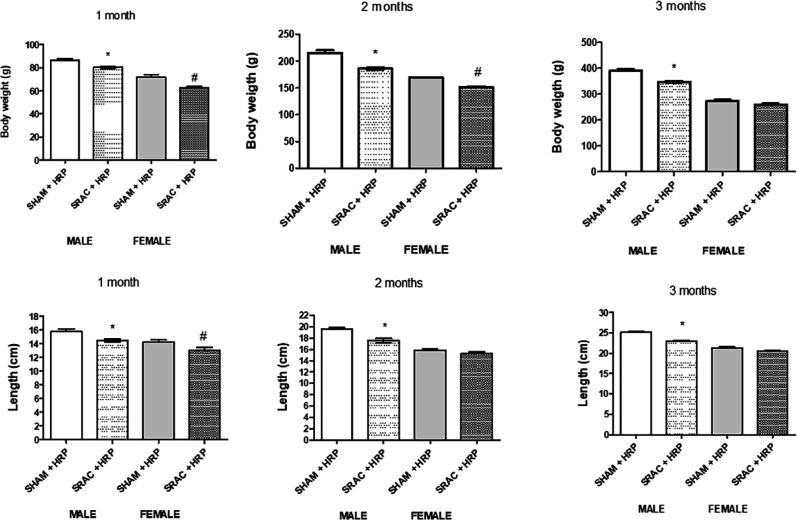
Body weight and length in offspring from normal (SHAM) and PE (SRAC) pregnancies with Handle region peptide (HRP) treatment

Treatment with HRP (Figure 3), induced a slightly higher recovery rate of both parameters; however, this recovery was not substantial. This seems to indicate that sexual maturity, probably with female hormone-induced changes, might contribute to a “normalization” of this parameter. 

**Figure 4:**
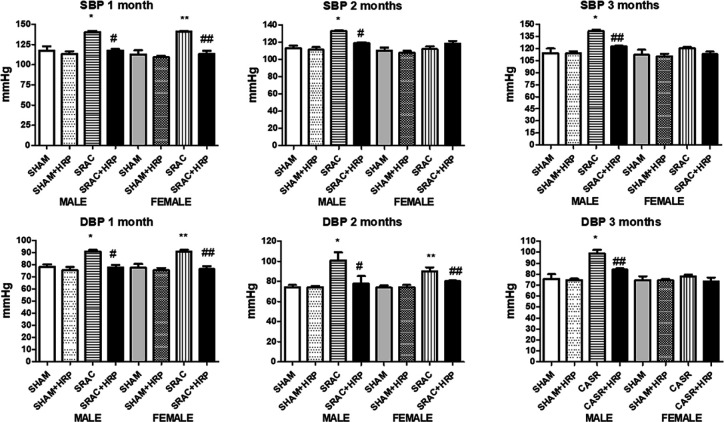
Blood pressure in offspring from normal (SHAM) and PE (SRAC) pregnancies with and without Handle region peptide (HRP) treatment

We measured blood pressure (BP) in both males and females after 4, 8, and 12 weeks after birth. As may be seen in Figure 4, one month-old normal offspring showed BP values ranging around 110 ± 4 mmHg in systolic and 73 ± 3 mmHg in diastolic pressures, both in males and females. Notoriously, more than 95 % of PE-derived animals showed high blood pressure, which remained throughout the experiment. In fact, systolic blood pressure was 140 ± 4 mmHg and diastolic was 94 ± 3 mmHg in both males and females. At 8 and 12 weeks, similarly to body weight, high blood remained elevated in males from PE compared to normal pregnancy-derived animals. These results strongly suggest that PE might induce illness in offspring, affecting mainly males. Once again, female animals showed lowering of blood pressure at these two periods of time, remarking the probable role of sexual hormones, which starts to increase in puberty. Puberty in rats is described beginning in weeks 8–9, which corresponds to our findings in markers described (21).

We also measured blood pressure in animals treated with HRP. As shown in Figure 4, systolic and diastolic blood pressures in normal animals with or without HRP were not modified. Whereas, pressures were elevated, both in male and female animals from PE pregnancies. HRP administration lowered systolic blood pressure in 4-week animals, from 140 ± 5 mmHg to 112 ± 3 mmHg in males; while in females, systolic blood pressure lowered from 141 ± 3 mmHg to 113 ± 4 mmHg. Regarding diastolic blood pressure, values in males and females were lowered from 91 ± 1 to 79 ± 2 mmHg and from 90 ± 2 to 79 ± 3 mmHg, respectively. At 8 weeks after birth, HRP diminished both systolic and diastolic blood pressures in males. In female animals, as we described above, hypertensive changes were not as evident as in males. HRP though, exerted a hypotensive effect only in diastolic pressure. Systolic blood pressure was not modified. At 12 weeks, only males remained hypertensive both in systolic and diastolic pressures, and HRP reverted these to normal values. Female animals were not hypertensive at this stage, and HRP did not show any effect upon blood pressure. These findings confirm that (P)RR seems to have a pro-hypertensive role, but also discovered a potential regulatory role of sexual female hormones on this effect.

**Figure 5:**
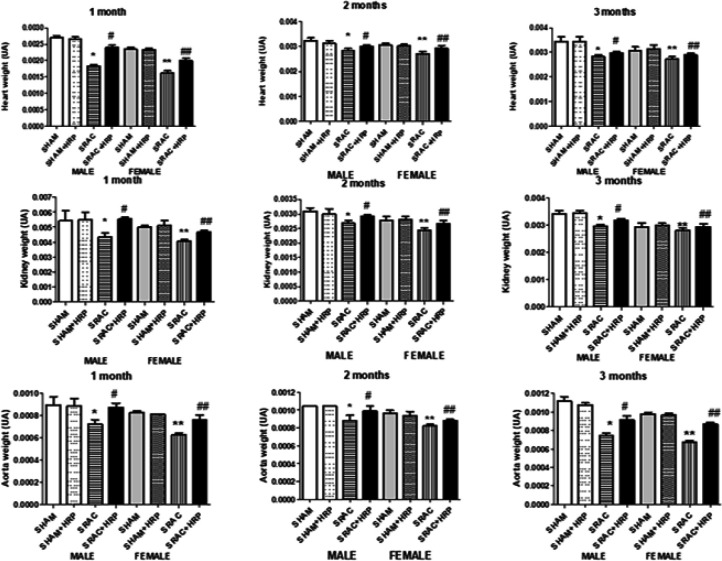
Heart, kidney, and aorta weight in offspring from normal (SHAM) and PE (SRAC) pregnancies with and without Handle region peptide (HRP) treatment

Because hypertension is a multi-systemic disease involving different organs, and after considering our weight results, we decided to measure the weight of different organs related to cardiovascular system, as heart and aorta, and kidneys in normal and PE-derived animals. In Figure 5 we show these results. We found that the three organs’ relative weight was different between control and PE-derived animals at all periods of time. Indeed, heart, aorta, and kidney weights were lower in both male and female animals delivered from PE pregnancies compared to normal pregnancy offspring. We did not find any recovery in any organ weight in females at 8 or 12 weeks. 

To evaluate participation of (pro)renin/renin receptor, a receptor of RAAS participating in hypertension, we administered HRP. This peptide forms part of the (pro)renin molecule that binds to (P)RR and is removed after conversion to renin. Ichihara developed this peptide as a competitive inhibitor for (P)RR, thus blocking its action (10). We administered 0.2 mg/Kg/day to similar groups of pups from PE pregnancies or from normal pregnancies. In Figure 5, the effect of HRP upon heart, aorta, and kidney weights, both in male and female animals is depicted. This peptide was able to increase weight in every organ after 7 days of treatment. In heart, HRP showed a marked increase in organ weight at 4 weeks after birth, from 0.001831 ± 0.00004 arbitrary units (AU) (SRAC group) to 0.0024 ± 0.00006 arbitrary units (AU) (SRAC + HRP) with *P*<0.005 in males; and from 0.001631 ± 0.00005 arbitrary units (AU) (SRAC group) to 0.001989 ± 0.00007 arbitrary units (AU) (SRAC + HRP) in females, with *P*<0.005. At 8 and 12 weeks however, increasing was not significant as in the first month, changing from 0.002844 ± 0.00008 arbitrary units (AU) (SRAC group) to 0.003004 ± 0.00006 arbitrary units (AU) (SRAC + HRP) in males, and from 0.002705 ± 0.0001 arbitrary units (AU) to 0.002938 ± .0001 arbitrary units (AU) in females at 8 weeks. At 12 weeks, change was also small. In both cases, weight recovery did not reach control values. Similar findings were obtained in aorta, and although weight recovery was higher in 8 and 12 weeks, this marker did not reach control values. Kidney showed heart-comparable changes: a marked increase at 4 weeks, but a modest increase after 8 and 12 weeks. These results suggest a possible participation of (P)RR upon organ development, and apparently gender does not influence this effect.

It has been described that (P)RR activates PLZF and the WNT signal transduction pathway, which, in turn, activates the canonical WNT pathway through β-catenin and DVL-1, and the non-canonical WNT pathway, through DVL-1 alone, and being PKCα a marker of activation of this pathway (22, 23). We explored these paths in 4, 8, and 12 weeks after birth animals in both males and females using immunoblot. 

**Figure 6:**
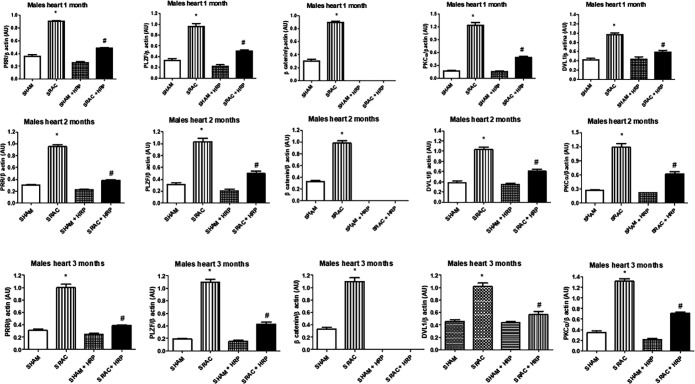
Immunoblot for PRR, PLZF, β-catenin, PKCα, and DVL-1 in male offspring from normal (SHAM) and PE (SRAC) pregnancies with and without Handle region peptide (HRP) treatment

**Figure 7:**
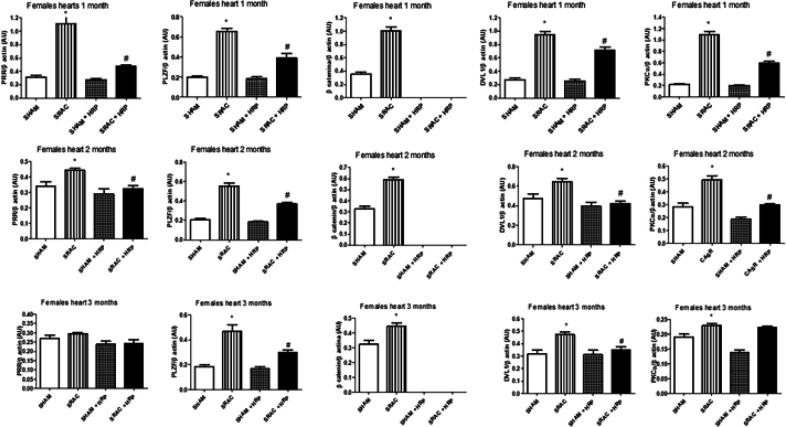
Immunoblot for PRR, PLZF, β-catenin, PKCα, and DVL-1 in female offspring from normal (SHAM) and PE (SRAC) pregnancies with and without Handle region peptide (HRP) treatment

In Figure 6 we show immunoblot results from male hearts and in Figure 7, from female hearts. We also ran kidney and aorta blots with similar results (data not shown). In same figures, we show the effect of HRP upon same proteins. We found a basal expression of (P)RR, PLZF, β-catenin, PKCα, and DVL-1 in hearts, kidneys, and aortas in both groups and at all ages explored. We found however, (P)RR expression increased importantly in animals from PE pregnancies in all tissues and ages. PLZF is an accepted marker of (P)RR activation (7). This protein also increased expression in all tissues and ages explored in PE-derived animals, both males and females, indicating a multi-place activation of this receptor. 

In same figure, we show that β-catenin was also increased significantly in the studied organs in both males and females with all ages tested. Interestingly, HRP treatment completely abolished this protein expression in these groups. This seems to indicate that HRP effect could be mediated by the canonical pathway. Indeed, DVL-1 and PKCα, proteins from the non-canonical pathway, were partially recovered in same groups, but did not reach basal levels. In this context and taking a closer look into gender differences, we found that the puberty-induced changes found in the other markers described above, seems to affect the canonical pathway and not the non-canonical pathway. As can be seen in the same figures, differences in DVL-1 and PKCα remain similar in male animals. β-catenin, on the other hand, showed a significant difference at 4 weeks of birth, was lower at 8 weeks, and there were no differences at 12 weeks in female animals in kidney and aorta (data not shown).

**Figure 8:**
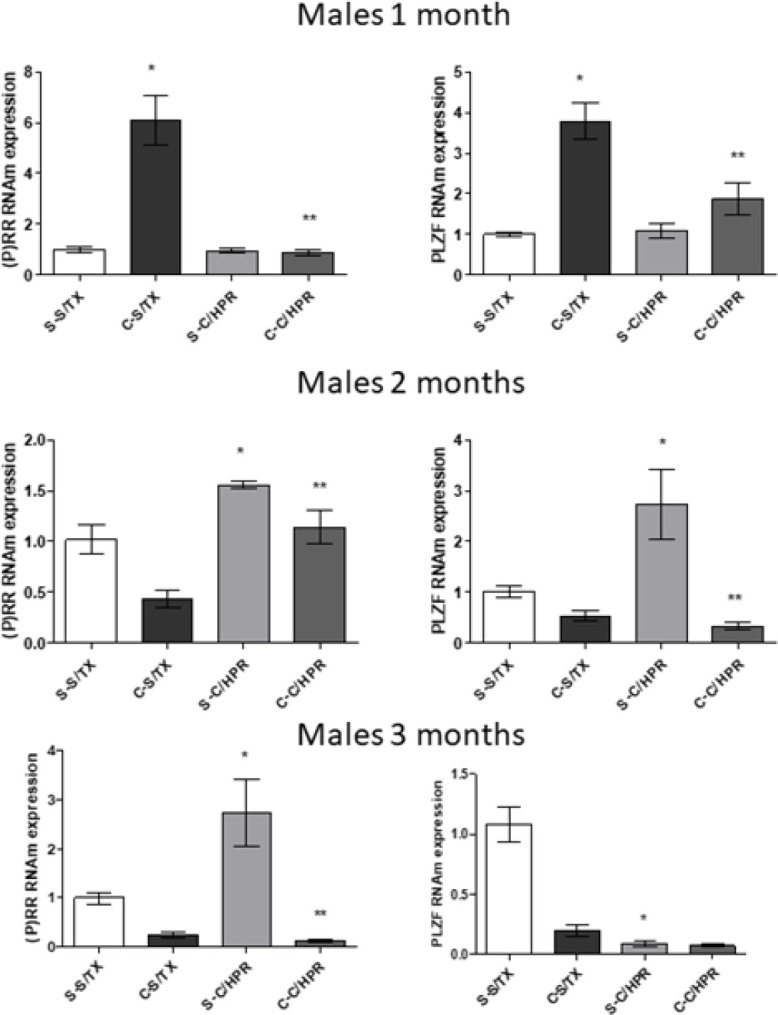
RT-PCR for PRR and PLZF in male offspring from normal (S) and PE (C) pregnancies with and without Handle region peptide (HRP) treatment

**Figure 9:**
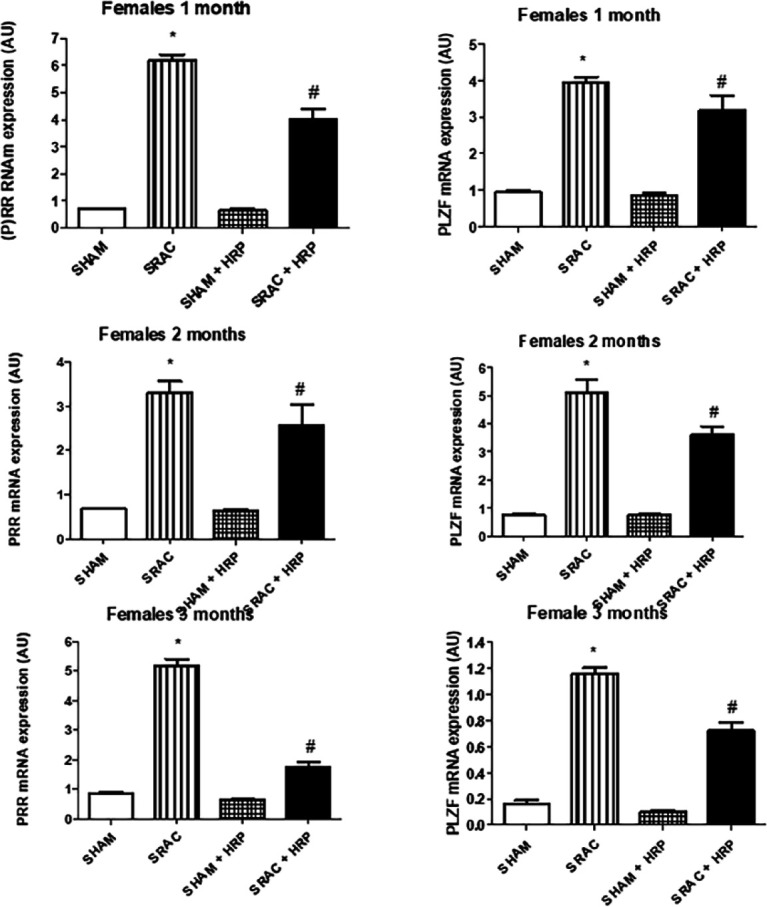
RT-PCR for PRR and PLZF in female offspring from normal (SHAM) and PE (SRAC) pregnancies with and without Handle region peptide (HRP) treatment

Finally, to explore if the effects upon PRR and PLZF involve genetic expression, we carried out RT-PCR experiments. As shown in Figures 8 and 9, (P)RR and PLZF expression increased in animals from PE pregnancies. HRP treatment reverted these changes in a similar way to the ones described for immunoblot experiments.

Following this, various elements of the beta-catenin destruction complex are brought to the membrane, hindering the phosphorylation of beta-catenin. As a result, beta-catenin can accumulate in the cytoplasm and move into the nucleus, where it associates with transcription factors to activate the transcription of WNT target genes like cyclin-D1, c-myc, and axin2. B. In the absence of Ang II, the (P)RR-prorenin triggers extracellular-signal-regulated kinase 1 (ERK1), ERK2, and p38 mitogen-activated protein kinase (MAPK) signaling pathways. These pathways, in turn, initiate the expression of genes associated with fibrosis and inflammation

**Table I:** PE model indicators: Blood pressure, proteinuria and intrauterine growth restriction

	NS	PE
Systolic pressure	100.3 ± 1.9	168.7 ± 6.5 *
Diastolic pressure	71.31 ± 2.1	118.4 ± 2.5 *
Proteinuria	0.7527 ± 0.042	1.364 ± 0.07440 *
Number of pups	14.75 ± 0.47	10 ± 0.7 *
Pup weight	6.8 ± 0.13	4.6 ± 0.17 *
Pup length	5.8 ± 0.1	4 ± 0.04 *

NS: Non SRAC, 3rd week pregnant rats. PE: SRAC, 3rd week pregnant rats. Groups were set with at least 4-6 rats. Systolic and diastolic blood pressure is expressed in mmHg; proteinuria is expressed in mg/mL, pup weight and length are expressed in g and cm, respectively. *P<0.05 compared to NS group.

SRAC: Subrenal aortic coarctation; PE: Preeclampsia

Animals were followed for 3 months. Results from male and female rats are shown. Body weight is expressed in g, length in cm. N = At least 6 animals per group. * *P*<0.05 compared to sham male group. # *P*<0.05 compared to sham female group

SRAC: Subrenal aortic coarctation; PE: Preeclampsia

HRP, 0.2 mg/kg/day, was administered subdermally for 7 days. Control group received same volume of 0.9 % saline. Animals were followed for 3 months. Both treatments were given last week of every month. Results from male and female rats are shown. Body weight is expressed in g, length in cm. N = At least 6 animals per group. * *P*<0.05 compared to sham male group. # *P*<0.05 compared to sham female group.

SRAC: Subrenal aortic coarctation; PE: Preeclampsia

Systolic (SBP) and diastolic (DBP) pressures are shown in mmHg. Animals were followed for 3 months. Results from male and female rats are shown. HRP was given as described in Figure 2. * *P*<0.05 compared to sham male group. # *P*<0.05 compared to SRAC male group. ** *P*<0.05 compared to sham female group. ## *P*<0.05 compared to SRAC female group.

SRAC: Subrenal aortic coarctation; PE: Preeclampsia

Organ weight was normalized using total body weight and shown as arbitrary units (AU). Animals were followed for 3 months. Results from male and female rats are shown. HRP was given as described in Figure 2. * P<0.05 compared to sham male group. # *P*<0.05 compared to SRAC male group. ** *P*<0.05 compared to sham female group. ## *P*<0.05 compared to SRAC female group.

SRAC: Subrenal aortic coarctation; PE: Preeclampsia

Heart (shown here), kidney and aorta (shown in supplemental material), from at least 4 males were normalized using β-actin as housekeeping protein. Samples were taken at the end of every month the experiment lasted. * *P*<0.05 compared to sham group. # *P*<0.05 compared to sham, HRP treated group.

SRAC: Subrenal aortic coarctation; PE: Preeclampsia

Heart, kidney, and aorta from at least 4 females were normalized using β-actin as housekeeping protein. Samples were taken at the end of every month the experiment lasted. * *P*<0.05 compared to sham group. # *P*<0.05 compared to sham, HRP treated group.

SRAC: Subrenal aortic coarctation; PE: Preeclampsia

Heart, kidney, and aorta from at least 4 males were taken and expression levels of the targeted mRNAs were normalized with 18S mRNA. Samples were taken at the end of every month the experiment lasted. * P<0.05 compared to sham group. ** *P*<0.05 compared to sham, HRP treated group.

PLZF: Promyelocytic leukaemia zinc finger protein; PRR: Pro renin/renin receptor

Heart, kidney, and aorta from at least 4 females were taken and expression levels of the targeted mRNAs were normalized with 18S mRNA. Samples were taken at the end of every month the experiment lasted. * *P*<0.05 compared to the sham group. # *P*<0.05 compared to sham, HRP treated group.

PLZF: Promyelocytic leukaemia zinc finger protein; PRR: Pro renin/renin receptor

## Discussion

In this work we showed that PE affects offspring in a severe way, inducing cardiovascular changes as hypertension and developmental problems. Interestingly, we found that these changes are partially mediated by RAAS, specifically (P)RR through the canonical WNT pathway, and that gender influences these changes.

PE belongs to a series of illnesses gathered under the name of pregnancy-induced hypertension and is relevant due to its negative effect upon mother and offspring (24). In mothers, it may induce several alterations after delivery. Of greater interest for us are the alterations that PE may induce in offspring. In fact, several morphological and physiological changes have been described (25). Among them, low birth weight and length are consistent changes. We found in our experiments that birth weight and length of pups were smaller than normal pregnancies offspring, both in male and female animals. Changes in these parameters have been described also in female mice (25); however, that recovery could not be related to gender. In our work, we found that male animals were not able to recover body weight even when they reached adult stage after 3 months. Female animals, on the other side, recovered both body weight and length at the third month, apparently due to sexual maturity. When we measured these parameters in animals under HRP treatment, we found a very slight change in both male and female animals, suggesting a possible role of (P)RR.

From the pioneer work from Barker, several authors have shown that PE and PE-induced low birth weight could affect offspring, especially males, by developing hypertension, metabolic syndrome, diabetes, and other morbidities (2, 3, 26). In our work, we found that both males and females suffered hypertension since the first month of age until the 3^rd^ month the experiment lasted. Along with it, they had low birth weight and length. We found that HRP treatment reverted this hypertension throughout the whole experiment in males. In females, the effect was also presented until the third month, where HRP had no more effect. This seems to confirm that (P)RR has a role in male hypertension. In females, it has a similar effect until, probably, sexual hormones normalize high blood pressure. This pro hypertensive role of (P)RR has been previously described(27); however, this is the first time that the effect is described in female animals and, even more, the effect of gender upon its role. 

It is also described that (P)RR participates in tissue maturity and development (12, 22). We described in offspring, both sexes, that heart, kidney, and aorta showed a clear reduction of organ weight caused by PE. When HRP was given, these reductions were consistently reverted on the three periods of time explored. Renin angiotensin aldosterone system (RAAS) has been widely related to development of cardiovascular, nervous, metabolic, and immunological illnesses (28, 29). Since (P)RR was described, it has been related to several pathological stages such as diabetic nephropathy, hypertension, and diabetes mellitus (22, 27, 30, 31). We have previously reported its prohypertensive role in PE when studied in pregnant rats (7). Our findings seem to point out that this receptor also possess a role in offspring illness, as we have shown with reduction of blood pressure values with HRP treatment, and corrections of morphological alterations in three different organs, all of them related to the hypertensive process. A very interesting finding in our work was the fact that, apparently, female sexual hormones seem to have a regulatory role upon its function.

We finally showed that (P)RR seems to activate the canonical WNT pathway to induce changes. Several pathways have been related to the function of this receptor: MAPK pathway, WNT pathway and acidification of intracellular media by activation of ATPase6AP2 (12, 32). WNT pathway is a biologically relevant pathway related to embryonic development, tissue homeostasis, injury repair and several diseases, such as cancer (33). Two main downstream paths have been described, both activated by (P)RR: Canonical and not canonical. The first one is activated by β-catenin. The second one is related more to DVL-1 and intracellular Ca^++^ movement, affecting cell polarity (34). We found that PE activates importantly β-catenin expression in three different tissues from PE-derived offspring throughout the whole experiment. Also, DVL-1 and PKCα are increased in both males and females at 1, 2, and 3 months after birth. However, and very interestingly, HRP treatment completely abolished β-catenin expression, while DVL-1 and PKCα remained elevated in same animals and same periods of time. PCR experiments for (P)RR and PLZF confirm that HRP effect seems to be at the genetic expression level. 

Several reports have related RAAS and WNT canonical pathway to cardiovascular pathologies, as hypertension and cardiac hypertrophy (14, 33). Inhibition of RAAS has also an inhibitory role upon WNT signaling, indicating a close interrelationship between these two systems (35). We found that HRP, acting as a (P)RR blocker, abolished β-catenin. This finding could explain the anti-hypertensive effect of the peptide in offspring from PE pregnancies. Given that WNT also have an injury repairing role, the reversal of morphological changes found in the heart, kidney, and aortas from PE-derived animals after HRP treatment could be explained by this action. It is necessary to mention that, regarding these transductional pathways, we did not find any modification related to gender or period of time. Although this does not rule out the possible role of age and gender, it strongly suggests that the effect could be explained by a more complex interaction of RAAS, specifically (P)RR, with other physiological systems as sexual hormones or another endocrine, cardiovascular, nervous, or immunological systems. More work is needed to answer these possibilities.

## Conclusion

We showed in this work that PE affects offspring, inducing cardiovascular alterations as hypertension and developmental problems. We also demonstrated, for the first time, that these changes might be mediated by (P)RR through the canonical WNT pathway, and that gender influences these changes.

## Authors’ Contributions

BP LG contributed to conceptualization, investigation, writing, data analysis, and visualization. CB S helped with investigation, visualization, and data analysis. R R helped in investigation in RT-PCR experiments, data analysis, and visualization. RT E provided methodology, resources, and validation. HC ME contributed by funding acquisition, conceptualization, writing, and supervision. LS P helped with conceptualization, funding acquisition, project administration, writing, formal analysis, and supervision.

## Conflicts of Interest

The authors declare that there are no conflicts of interest.

## References

[1] Fox R, Kitt J, Leeson P, Aye CYL, Lewandowski AJ (2019). Preeclampsia: Risk factors, diagnosis, management, and the cardiovascular impact on the offspring. J Clin Med.

[2] Barker DJ (2007). The origins of the developmental origins theory. J Intern Med.

[3] Cheong JN, Wlodek ME, Moritz KM, Cuffe JS (2016). Programming of maternal and offspring disease: Impact of growth restriction, fetal sex and transmission across generations. J Physiol.

[4] Paauw ND, van Rijn BB, Lely AT, Joles JA (2017). Pregnancy as a critical window for blood pressure regulation in mother and child: Programming and reprogramming. Acta Physiol (Oxf).

[5] Vo T, Hardy DB (2012). Molecular mechanisms underlying the fetal programming of adult disease. J Cell Commun Signal.

[6] Ligi I, Grandvuillemin I, Andres V, Dignat-George F, Simeoni U (2010). Low birth weight infants and the developmental programming of hypertension: A focus on vascular factors. Semin Perinatol.

[7] Avila-Ramírez MA, Esteban-Martínez RL, López-Moctezuma E, Anguiano-Robledo L, Hernández-Campos ME, López-Sánchez P (2019). (Pro)renin/renin receptor expression during normal and preeclamptic pregnancy in rats. Life Sci.

[8] Hennrikus M, Gonzalez AA, Prieto MC (2018). The prorenin receptor in the cardiovascular system and beyond. Am J Physiol Heart Circ Physiol.

[9] Intapad S, Ojeda NB, Varney E, Royals TP, Alexander BT (2015). Sex-specific effect of endothelin in the blood pressure response to acute angiotensin II in growth-restricted rats. Hypertension.

[10] Ichihara A, Hayashi M, Kaneshiro Y, Suzuki F, Nakagawa T, Tada Y (2004). Inhibition of diabetic nephropathy by a decoy peptide corresponding to the “handle” region for nonproteolytic activation of prorenin. J Clin Invest.

[11] Ichihara A, Suzuki F, Nakagawa T, Kaneshiro Y, Takemitsu T, Sakoda M (2006). Prorenin receptor blockade inhibits development of glomerulosclerosis in diabetic angiotensin II type 1a receptor-deficient mice. J Am Soc Nephrol.

[12] Nguyen G, Muller DN (2010). The biology of the (pro)renin receptor. J Am Soc Nephrol.

[13] Vallee A, Levy BL, Blacher J (2018). Interplay between the renin-angiotensin system, the canonical WNT/beta-catenin pathway and PPARgamma in hypertension. Curr Hypertens Rep.

[14] Badimon L, Borrell-Pages M (2017). Wnt signaling in the vessel wall. Curr Opin Hematol.

[15] Anguiano-Robledo L, Reyes-Melchor PA, Bobadilla-Lugo RA, Perez-Alvarez VM, Lopez-Sanchez P (2007). Renal angiotensin-II receptors expression changes in a model of preeclampsia. Hypertens Pregnancy.

[16] Abitbol MM (1982). Simplified technique to produce toxemia in the rat: considerations on cause of toxemia. Clin Exp Hypertens B.

[17] Podjarny E, Losonczy G, Baylis C (2004). Animal models of preeclampsia. Semin Nephrol.

[18] Li J, LaMarca B, Reckelhoff JF (2012). A model of preeclampsia in rats: The reduced uterine perfusion pressure (RUPP) model. Am J Physiol Heart Circ Physiol.

[19] Javadian P, Salmanian B, Javadi-Paydar M, Shamshirsaz AA, Ejtemaei Mehr S, Gharedaghi MH (2013). Effect of morphine on the reduced uteroplacental perfusion model of pre-eclampsia in rats. Eur J Obstet Gynecol Reprod Biol.

[20] Ramirez-Montero C, Lima-Gomez V, Anguiano-Robledo L, Hernandez-Campos ME, Lopez-Sanchez P (2020). Preeclampsia as predisposing factor for hypertensive retinopathy: Participation by the RAAS and angiogenic factors. Exp Eye Res.

[21] Sengupta P (2013). The laboratory rat: Relating its age with human’s. Int J Prev Med.

[22] Sihn G, Rousselle A, Vilianovitch L, Burckle C, Bader M (2010). Physiology of the (pro)renin receptor: Wnt of change?. Kidney Int.

[23] Li C, Siragy HM High glucose induces podocyte injury via enhanced (pro)renin receptor-Wnt-beta-catenin-snail signaling pathway. PLoS One.

[24] Kintiraki E, Papakatsika S, Kotronis G, Goulis DG, Kotsis V (2015). Pregnancy-induced hypertension. Hormones (Athens).

[25] Sutton EF, Lob HE, Song J, Xia Y, Butler S, Liu CC (2017). Adverse metabolic phenotype of female offspring exposed to preeclampsia in utero: A characterization of the BPH/5 mouse in postnatal life. Am J Physiol Regul Integr Comp Physiol.

[26] Calkins K, Devaskar SU (2011). Fetal origins of adult disease. Curr Probl Pediatr Adolesc Health Care.

[27] Ramkumar N, Kohan DE (2019). The (pro)renin receptor: An emerging player in hypertension and metabolic syndrome. Kidney Int.

[28] Takimoto-Ohnishi E, Murakami K (2019). Renin-angiotensin system research: From molecules to the whole body. J Physiol Sci.

[29] Miller AJ, Arnold AC (2019). The renin-angiotensin system in cardiovascular autonomic control: Recent developments and clinical implications. Clin Auton Res.

[30] Ichihara A, Kaneshiro Y, Takemitsu T, Sakoda M, Nakagawa T, Nishiyama A (2006). Contribution of nonproteolytically activated prorenin in glomeruli to hypertensive renal damage. J Am Soc Nephrol.

[31] Ichihara A, Kaneshiro Y, Takemitsu T, Sakoda M, Suzuki F, Nakagawa T (2006). Nonproteolytic activation of prorenin contributes to development of cardiac fibrosis in genetic hypertension. Hypertension.

[32] Jan Danser AH, Batenburg WW, van Esch JH (2007). Prorenin and the (pro)renin receptor--an update. Nephrol Dial Transplant.

[33] Zhao Y, Wang C, Wang C, Hong X, Miao J, Liao Y (2018). An essential role for Wnt/beta-catenin signaling in mediating hypertensive heart disease. Sci Rep.

[34] Habas R, Dawid IB (2005). Dishevelled and Wnt signaling: is the nucleus the final frontier?. J Biol.

[35] Zhou L, Li Y, Hao S, Zhou D, Tan RJ, Nie J (2015). Multiple genes of the renin-angiotensin system are novel targets of Wnt/beta-catenin signaling. J Am Soc Nephrol.

